# Non-invasive assessment of stimulation-specific changes in cerebral glucose metabolism with functional PET

**DOI:** 10.1007/s00259-024-06675-0

**Published:** 2024-03-16

**Authors:** Godber Mathis Godbersen, Pia Falb, Sebastian Klug, Leo R. Silberbauer, Murray Bruce Reed, Lukas Nics, Marcus Hacker, Rupert Lanzenberger, Andreas Hahn

**Affiliations:** 1 Department of Psychiatry and Psychotherapy, Medical University of Vienna, Vienna, Austria; 2 Comprehensive Center for Clinical Neurosciences and Mental Health (C3NMH), Medical University of Vienna, Vienna, Austria; 3 Department of Biomedical Imaging and Image-Guided Therapy, Division of Nuclear Medicine, Medical University of Vienna, Vienna, Austria

**Keywords:** Brain metabolism, Functional PET (fPET), Quantification, Percent signal change, Cerebral metabolic rate of glucose (CMRGlu)

## Abstract

**Purpose:**

Functional positron emission tomography (fPET) with [^18^F]FDG allows quantification of stimulation-induced changes in glucose metabolism independent of neurovascular coupling. However, the gold standard for quantification requires invasive arterial blood sampling, limiting its widespread use. Here, we introduce a novel fPET method without the need for an input function.

**Methods:**

We validated the approach using two datasets (DS). For DS1, 52 volunteers (23.2 ± 3.3 years, 24 females) performed Tetris® during a [^18^F]FDG fPET scan (bolus + constant infusion). For DS2, 18 participants (24.2 ± 4.3 years, 8 females) performed an eyes-open/finger tapping task (constant infusion). Task-specific changes in metabolism were assessed with the general linear model (GLM) and cerebral metabolic rate of glucose (CMRGlu) was quantified with the Patlak plot as reference. We then estimated simplified outcome parameters, including GLM beta values and percent signal change (%SC), and compared them, region and whole-brain-wise.

**Results:**

We observed higher agreement with the reference for DS1 than DS2. Both DS resulted in strong correlations between regional task-specific beta estimates and CMRGlu (*r* = 0.763…0.912). %SC of beta values exhibited strong agreement with %SC of CMRGlu (*r* = 0.909…0.999). Average activation maps showed a high spatial similarity between CMRGlu and beta estimates (Dice = 0.870…0.979) as well as %SC (Dice = 0.932…0.997), respectively.

**Conclusion:**

The non-invasive method reliably estimates task-specific changes in glucose metabolism without blood sampling. This streamlines fPET, albeit with the trade-off of being unable to quantify baseline metabolism. The simplification enhances its applicability in research and clinical settings.

**Supplementary Information:**

The online version contains supplementary material available at 10.1007/s00259-024-06675-0.

## Introduction

Functional positron emission tomography (fPET) using the radiolabeled glucose analogue 2-[^18^F]-fluorodeoxyglucose ([^18^F]FDG) holds significant promise for investigating the dynamics of brain metabolism [1]. Using constant infusion of the radiotracer, fPET enables the assessment of changes in metabolic demands in response to external stimulation, such as cognitive tasks [2–4] within a single PET scan. Furthermore, these dynamics are independent from cerebral blood flow and neurovascular coupling [2] and the neuronal activation based on glucose metabolism can be absolutely quantified [3]. Moreover, the widespread availability of [^18^F]FDG and the compatibility with standard PET scanners make fPET an easily accessible tool for functional neuroimaging. However, a major drawback limiting its widespread use is the need for arterial blood samples during the scan to determine the cerebral metabolic rate of glucose (CMRGlu).

The gold standard for absolute quantification in PET imaging relies on the arterial input function (AIF). However, arterial cannulation has inherent disadvantages. These include the need for skilled physicians, increased experimental complexity as well as patient discomfort or pain and in rare cases potential complications [5]. These limitations raise the question of whether task-specific changes in glucose metabolism using fPET can be obtained without arterial blood sampling.

Several alternatives to obtain an AIF have been proposed for fPET. Venous samples [2, 3, 6] have been shown to yield sufficiently accurate quantification if the radiotracer is administered only via constant infusion [3]. However, such a protocol results in low signal-to-noise ratio (SNR), which, among others, affects accuracy in movement correction and quantification of task effects. The use of an initial bolus resolves these issues [7]. However, by adding a bolus, venous samples may not be adequate anymore due to a delay in the equilibration between blood pools and the subsequent underestimation of the area under the curve. The use of population-based input functions [8] is another option that avoids blood sampling. However, the assumption of equal pharmacokinetics across participants makes the approach susceptible to individual variation [9]. Image-derived input functions (IDIFs) represent another option [10], but robust extraction from large blood pools may be limited to large field of view PET scanners. In sum, the mentioned alternatives to AIF offer easier applicability at the expense of accuracy, but may not fully eliminate the need for blood sampling.

To resolve this issue, we evaluate the feasibility of quantifying task-induced metabolic demands using [^18^F]FDG fPET without any blood sampling. We hypothesize that the input function can be omitted when task-specific activation is the primary outcome of interest. This is because the general linear model (GLM) readily separates task effects from baseline metabolism, thus yielding task-specific estimates for activation. By eliminating the requirement for blood sampling, we aim to simplify both acquisition and processing thus increasing the accessibility of fPET.

## Materials and methods

### Mathematical rationale

Our proposition that the GLM may be adequate for evaluating task-specific changes in glucose metabolism is grounded in the following mathematical rationale. For irreversibly binding radiotracers such as [^18^F]FDG, the ratio of tracer concentration in tissue C_T_ to that in plasma C_P_ at a certain time point t can be characterized using the Patlak plot [11]:

1 $$\frac{{C}_{T}(t)}{{C}_{P}(t)}={K}_{i}\frac{{\int }_{0}^{t}{C}_{P}\left(\tau \right)d\tau }{{C}_{P}(t)}+intercept$$

The net influx constant *K*_*i*_ is the estimated outcome parameter, which is determined as the slope of the Patlak plot when it approaches linearity after t* [11]. The absolute amount of CMRGlu is then determined by:

2 $$CMRGlu={K}_{i}\frac{{Glu}_{P}}{LC}*100$$

LC refers to the lumped constant and Glu_P_ represents the concentration of glucose in plasma. Rearrangement of Eq. (1) yields

3 $${K}_{i}=\frac{{C}_{T}\left(t\right)-{\text{intercept}}*{C}_{P}(t)}{{\int }_{0}^{t}{C}_{P}\left(\tau \right)d\tau }$$

Assuming that intercept * *C*_*P*_ <  < *C*_*T*_, the relation reduces to

4 $${K}_{i}=\frac{{C}_{T}\left(t\right)}{{\int }_{0}^{t}{C}_{P}\left(\tau \right)d\tau }$$

This assumption is particularly true for a small bolus and after linearity of the Patlak plot has already been reached. The parameter in Eq. (4) is identical to the previously described “fractional uptake of [^18^F]FDG normalized by plasma activity” [12]. This normalization stems from the inherent mathematical assumptions made while deriving the relation. Furthermore, in the relationship between task effects and baseline metabolism, the integral of the plasma concentration also cancels out. This implies that the ratio between the tissue concentrations is directly proportional to the relative changes in *K*_*i*_ (and thus relative changes in CMRGlu, see Eq. (2))

5 $$\%SC\propto \frac{{K}_{i, task}}{{K}_{i, BL}}\propto \frac{{C}_{T, task}(t)}{{C}_{T, BL}(t)}\propto \frac{{\beta }_{task}*{regressor}_{task}(t)}{{\beta }_{BL}*{regressor}_{BL}(t)}$$

In this equation, β represents the output of the GLM when the respective regressors are used for modeling. Consequently, the ratio of the GLM’s output modeling task and baseline effects should also vary proportionally to the relative changes in CMRGlu. Since the multiplication of a beta value with its corresponding regressor represents a time course, we estimated its slope for the computation of percent signal changes (%SC, see “Surrogate parameters”). This approach was chosen because different regressors were used for task and baseline in the GLM, which implies that simple beta values cannot be directly compared.

### In vivo datasets

**Fig. 1:**
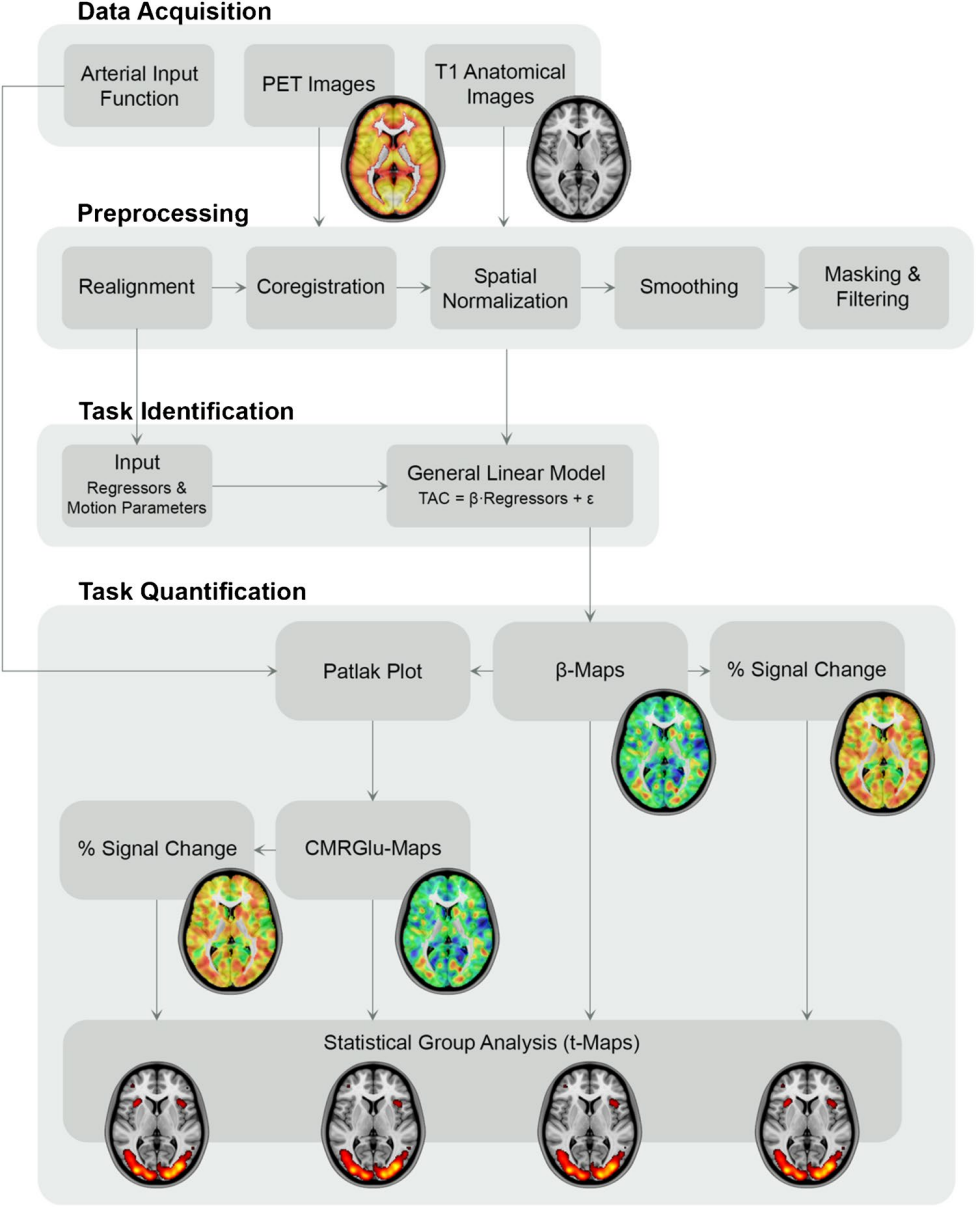
Schematic workflow of the preprocessing and analysis routine. After data acquisition and preprocessing, data were entered into a general linear model to separate task effects from baseline metabolism. Four different outcome parameters were then calculated: (i) the plain β-maps obtained from the general linear model and (ii) % signal change relative to baseline. Absolute quantification with the arterial input function and the Patlak plot yielded (iii) maps of cerebral rate of glucose metabolism (CMRGlu) and (iv) % signal change thereof

In order to test the hypothesis, we analyzed two separate datasets with different designs (Supplementary Fig. 1), tasks, and activated regions of interest (ROIs). For both datasets, similar methods of preprocessing and statistical analysis were applied. A schematic overview of the procedure is given in Fig. 1 and further details are provided in the experimental design and tasks section of the supplement.

The first dataset (DS1) includes simultaneous fPET/fMRI examinations in 52 healthy participants performing a challenging visuo-spatial motor coordination task in two levels of difficulty (modified version of Tetris®). After an initial baseline of eight minutes, each task level was performed two times and six minutes each, followed by five minutes of rest (Supplementary Fig. 1). Detailed descriptions of the design, acquisition and analysis are provided in our previous work [13], below and in the supplement.

The second dataset (DS2) comprises data of 18 healthy participants. The fPET/fMRI scan started with a baseline of 10 min. Afterwards, participants either tapped their right thumb to their other fingers (10–20 min and 60–70 min) or opened their eyes (35–45 min and 85–95 min). Details can be found in our previous work [14], below and in the supplement.

### Participants

DS1 includes 52 healthy participants (23.2 ± 3.3 years, 24 females, all right-handed), who were partly also included in previous work [13, 15–17]. DS2 comprises 18 healthy participants’ data (24.2 ± 4.3 years, 8 females, all right-handed), of which 15 had previously contributed to another study [3]. See supplement for details.

### PET data acquisition and processing

All fPET measurements were performed on the same fully integrated PET/MR system (Siemens mMR Biograph, Erlangen, Germany). Administration of [^18^F]FDG was done according to a bolus plus constant infusion protocol for DS1 and with constant infusion only for DS2. This enables the assessment of the performance of both administration protocols. Data pre-processing of both studies’ fPET data was done with SPM12 and included motion correction, spatial normalization to MNI-space and smoothing. For both datasets manual arterial blood samples were collected to construct the AIF. See supplement for details.

### Quantification of CMRGlu

In order to analyze task activation within the two datasets, a general linear model (GLM) was applied. Both models included one regressor for baseline, one for movement artifacts and two regressors associated with task activation. For DS1, these regressors referred to the two levels of task difficulty. For DS2, they represented the separate tasks of eyes-open and right finger-tapping (see supplement).

For the calculation of the respective influx constants (K_i_), the relevant Patlak plots were constructed and their respective slopes were identified as in Eq. (1). The start of the linear fit for the Patlak plot was set to approximately a third of the total scan time for both datasets, t* = 15 min for DS1 and t* = 30 min for DS2. The absolute quantification of CMRGlu was conducted in accordance with Eq. (2) and a value for the LC of 0.89, in both cases [18, 19]. The amount of CMRGlu was quantified in units of µmol/100 g/min.

### Surrogate parameters

Our primary goal was to obtain a metric that enables the identification of task-specific changes in glucose metabolism without invasive blood sampling. Thus, we compared four different parameters of interest: (i) the absolutely quantified values for CMRGlu (see Eq. (2)), used as the gold standard, (ii) the plain beta values calculated by the GLM, and (iii–iv) the percent signal change (%SC) of both quantities in relation to the baseline condition (see Eq. (5)). Thereby, we established a relationship between the beta values and CMRGlu as well as %SC of betas with %SC of CMRGlu. The %SC of CMRGlu was calculated as the ratio of task effects to baseline metabolism multiplied by 100. The %SC for the beta values cannot be directly retrieved from the GLM output since the betas are associated with different regressors. Consequently, the slopes of the time activity curves were estimated (in kBq/frame), represented by beta*regressor separately for task and baseline metabolism (see Eq. (5)). For the extraction of the slope of the baseline TAC, a linear fitting procedure was performed. The fit was applied for a similar time interval as for the Patlak plots. Specifically, for DS1 the linear fit started from minute 16 after the beginning of the radiotracer application until the end of the PET scan. For DS2, the interval began later due to the absence of an initial bolus, specifically from 30 min after the beginning until the end. Since the task regressors were modeled as ramp functions with a slope of 1 kBq/frame, the beta values for the tasks are already equivalent to the slope we aimed to extract. Hence, %SC of betas was then calculated as the ratio of the task and baseline slopes multiplied by 100.

Furthermore, two different baseline metrics (BL, BL2) were considered. Notably, for BL and BL2, no %SC data could be calculated, as the percent signal change inherently refers to the baseline condition itself. BL simply represents the beta value of the baseline condition as calculated by the GLM. BL2 was determined by calculating the slope of the curve given by multiplying the baseline regressor with the corresponding baseline beta values, i.e., a linear fit to the baseline beta * baseline regressor. We opted for the second baseline metric because this calculation also enters the determination of %SC of the beta values, allowing for a direct comparison. Furthermore, BL2 takes the individual variation in the baseline regressor into account and is therefore comparable across participants. It is worth noting that BL is also identical to standardized uptake value ratios (SUVR) with reference to global tracer uptake. That is, regional tracer uptake is represented by regional baseline beta * baseline regressor [3] and since the baseline regressor represents the global tracer uptake, this cancels out when computing the ratio.

### Statistical analysis

The ROI analysis focused on the respective regions of significant activation (all *p* < 0.05 FWE corrected) for each dataset as obtained by group-level statistical analysis in our previous work. More precisely, for DS1, the regions selected for further investigation were the frontal eye field (FEF), the intraparietal sulcus (IPS), and the secondary occipital cortex (Occ). These were identified to be active in our previous work across three different functional approaches (fPET, BOLD, ASL) [16]. For DS2, the relevant ROIs were the primary occipital cortex (V1) as well as the primary motor cortex (M1), since these displayed significant task activation in our previous study [14]. Outcome parameters were then extracted for these ROIs and linear regression analysis was then performed for each pair of parameters using MATLAB R2018b.

For the voxel-wise analysis, group-level statistics were computed in SPM12 and a one-sample *t*-test was performed for each of the four parameters. Activation maps were extracted (all *p* < 0.05 FWE corrected cluster level following *p* < 0.001 uncorrected voxel level) and activation patterns across different approaches were compared using the Dice coefficient. For DS1, we extracted respective activation maps for the hard task difficulty and for DS2 for both open eyes and right finger-tapping tasks.

## Results

### Region of interest analysis

For DS1, we observed strong associations between GLM beta values and gold standard CMRGlu values for task-specific estimates of activation in the FEF, IPS and Occ (*r* = 0.833…0.912, Table 1). Crucially, near perfect correlations were discovered when comparing %SC of beta values with %SC of CMRGlu (all *r ≥* 0.998). Moreover, the slopes were close to unity (1.00…1.02) and intercepts were near zero (− 0.27…0.13) for the parameters of %SC. Separate analyses for females and males did not change any of these observations (Supplementary Table 1, Supplementary Fig. 2). DS2 showed similar results for task-related changes in glucose metabolism (eyes open and finger-tapping tasks, activating V1 and M1, respectively), albeit with slightly lower performance compared to DS1. Specifically, the correlation coefficients were higher for %SC (*r* = 0.909… 0.970) than beta values (*r* = 0.763…0.833). The slopes for %SC were close to one (1.03…1.15), but intercepts were slightly higher (1.63…4.73).

**Table 1:** Agreement between different quantification methods. The table displays the results of correlation and regression analyses conducted for both datasets. Comparisons were performed for two different levels, either relating the general linear model beta values to the respective cerebral metabolic rate of glucose (CMRGlu, left) or the percent signal change (%SC) of both quantities with each other (right). The first dataset (DS1) comprised three regions of interest (ROI): the frontal eye field (FEF), intraparietal sulcus (IPS), and occipital cortex (Occ) [16]. For these regions, two separate levels of task difficulty (easy, hard) were regarded. For the second dataset (DS2), the primary visual (V1), and motor cortices (M1) were evaluated during the eyes-open condition and right-finger-tapping task, respectively [14]. For all datasets, two approaches for the computation of baseline metabolism (BL and BL2) were calculated. For each comparison, Pearson’s correlation coefficient, slope and intercept were calculated. For the baseline conditions, the %SC analyses were not performed, as this parameter always refers to the baseline condition itself

Condition	ROI	Beta vs. CMRGlu			%SC of Beta vs. %SC of CMRGlu		
		*R*	Slope	Intercept	*R*	Slope	Intercept
BL	FEF	0.483	0.005	1.107			
	IPS	0.720	0.008	0.992			
	Occ	0.359	0.005	1.041			
	V1	0.018	0.000	1.137			
	M1	− 0.137	− 0.001	1.043			
BL2	FEF	0.466	0.005	0.137			
	IPS	0.504	0.005	0.131			
	Occ	0.433	0.004	0.142			
	V1	0.398	0.002	0.140			
	M1	0.337	0.002	0.131			
Tetris Easy	FEF	0.904	0.009	0.006	0.999	0.997	0.134
	IPS	0.857	0.008	0.007	0.998	1.001	0.053
	Occ	0.912	0.010	0.001	0.999	1.004	0.011
Tetris Hard	FEF	0.849	0.009	0.007	0.998	1.001	0.094
	IPS	0.833	0.008	0.008	0.998	1.003	0.028
	Occ	0.843	0.010	0.001	0.998	1.022	-0.273
Eye	V1	0.833	0.007	0.008	0.970	1.150	1.633
Finger	M1	0.763	0.005	0.016	0.909	1.029	4.728

In contrast, the baseline condition (BL) displayed a highly variable degree of association with CMRGlu, with *r* = 0.359…0.720 for DS1 and *r* =  − 0.137…0.018 for DS2. Although BL2 resulted in more stable agreement, correlations with CMRGlu were still low (*r* = 0.337…0.504).

### Voxel-wise activation maps

In addition to the ROI analysis, we conducted an unbiased whole-brain analysis to explore whether the different approaches yield similar activation patterns (all *p* < 0.05 FWE corrected cluster level following *p* < 0.001 uncorrected voxel level).

**Fig. 2:**
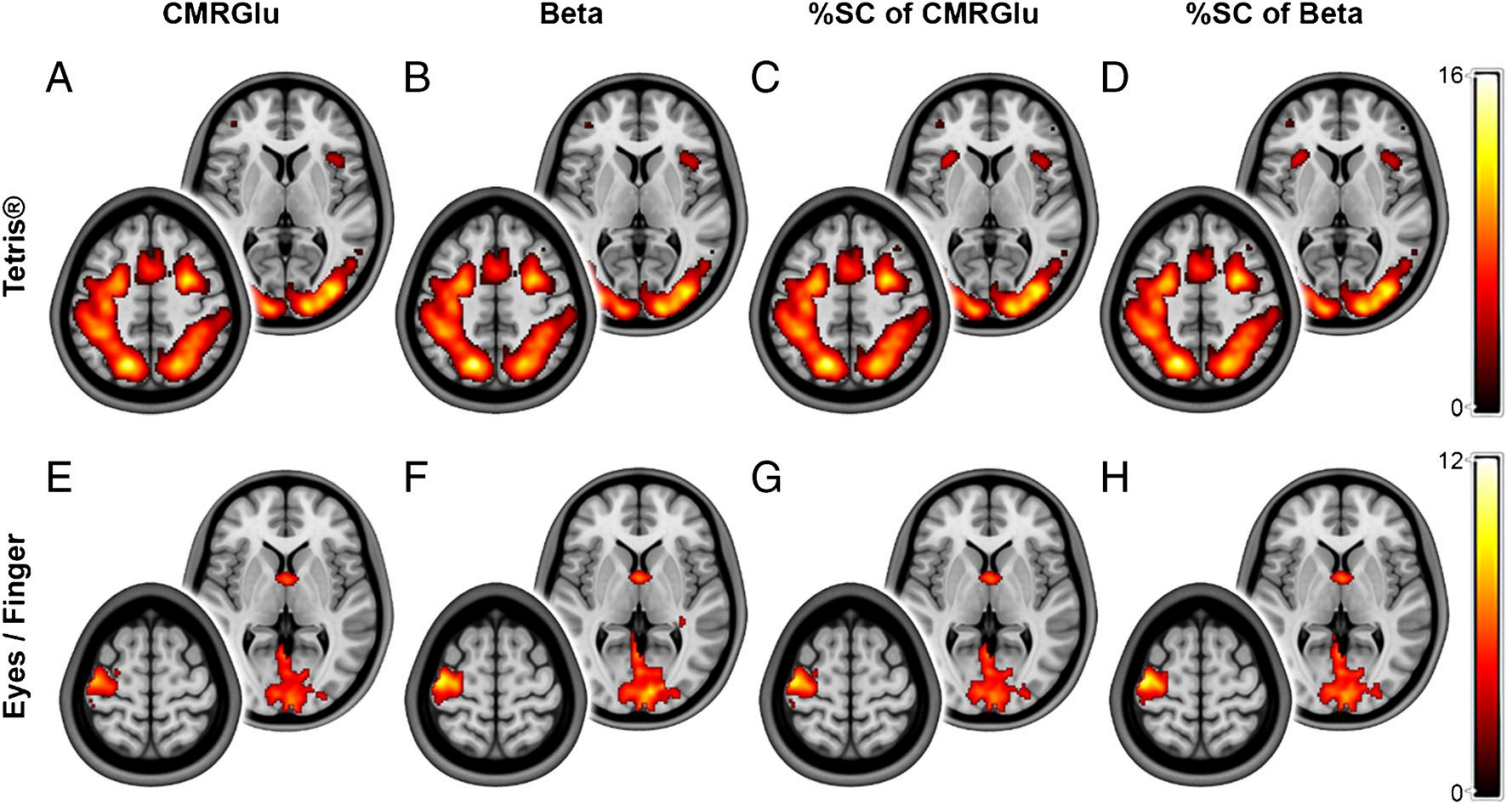
Group-level maps of the datasets, displaying activation within the respective regions of interest (ROI). The figure displays the activation patterns for both of the regarded datasets, considering task “hard” for the Tetris®-dataset (DS1, **A**–**D**), and both tasks within the second dataset (DS2, **E**–**H**). The maps were *p* < 0.05 FWE corrected at cluster level following *p* < 0.001 uncorrected voxel level. Group-level maps were calculated for the beta parameters (**B**, **F**), resulting from the general linear model (GLM), and the cerebral metabolic rate of glucose (CMRGlu, **A**, **E**) as well as for both quantities’ rate of percent signal change (%SC, **C**, **D**, **G**, **H**). For each of the group-level maps, two layers were selected to represent the activation within the respective dataset. For DS1 (**A**–**D**), the figure displays layers extracted at *z* = 6 mm (right) and *z* = 50 mm (left). For DS2 (**E**–**H**), the regarded layers are *z* = 3 mm (right) and *z* = 63 mm (left). The color bars represent t-values of the group-level analysis

As in our previous work [13, 16], task-related changes in CMRGlu were observed mainly in the FEF, IPS and Occ for DS1 (Fig. 2A). Interestingly, this was also true for all of the other parameters, namely maps representing beta values as well as %SC of beta and %SC of CMRGlu (Fig. 2B–D). For the easy and hard levels of difficulty, the dice coefficients of the beta maps and their respective CMRGlu counterparts amounted to 0.972 and 0.979, indicating high similarity. In accordance with the ROI results, comparing the %SC maps yielded even higher dice coefficients of 0.997 (for both conditions) between the parameters. Visual comparison of the outcome parameters at the individual level highlights the similarity within a subject as well as differences between subjects (see discussion and Supplementary Fig. 3).

For DS2, task-induced changes in CMRGlu occurred within V1 and M1 for the eyes open and finger-tapping tasks, respectively [14] (Fig. 2E). Again, the activation patterns for each of the two tasks were remarkably similar across all parameters (Fig. 2F–H). The dice coefficients of the beta and CMRGlu maps for the eyes open and finger-tapping tasks amounted to 0.885 and 0.870, respectively. The %SC data yielded the coefficients 0.943 and 0.932.

## Discussion

In this work, we evaluated the feasibility of non-invasively quantifying task-induced changes in glucose metabolism with [^18^F]FDG fPET, i.e., without any blood sampling. We integrated theoretical concepts from the Patlak plot with output parameters of the GLM. Moreover, we compared this with CMRGlu quantified with the gold standard arterial input function in various tasks. Our findings reveal similar activation patterns across all parameters and strong agreement between the relative changes of glucose metabolism (%SC CMRGlu) and task-specific beta values obtained from the GLM (%SC betas).

**Fig. 3:**
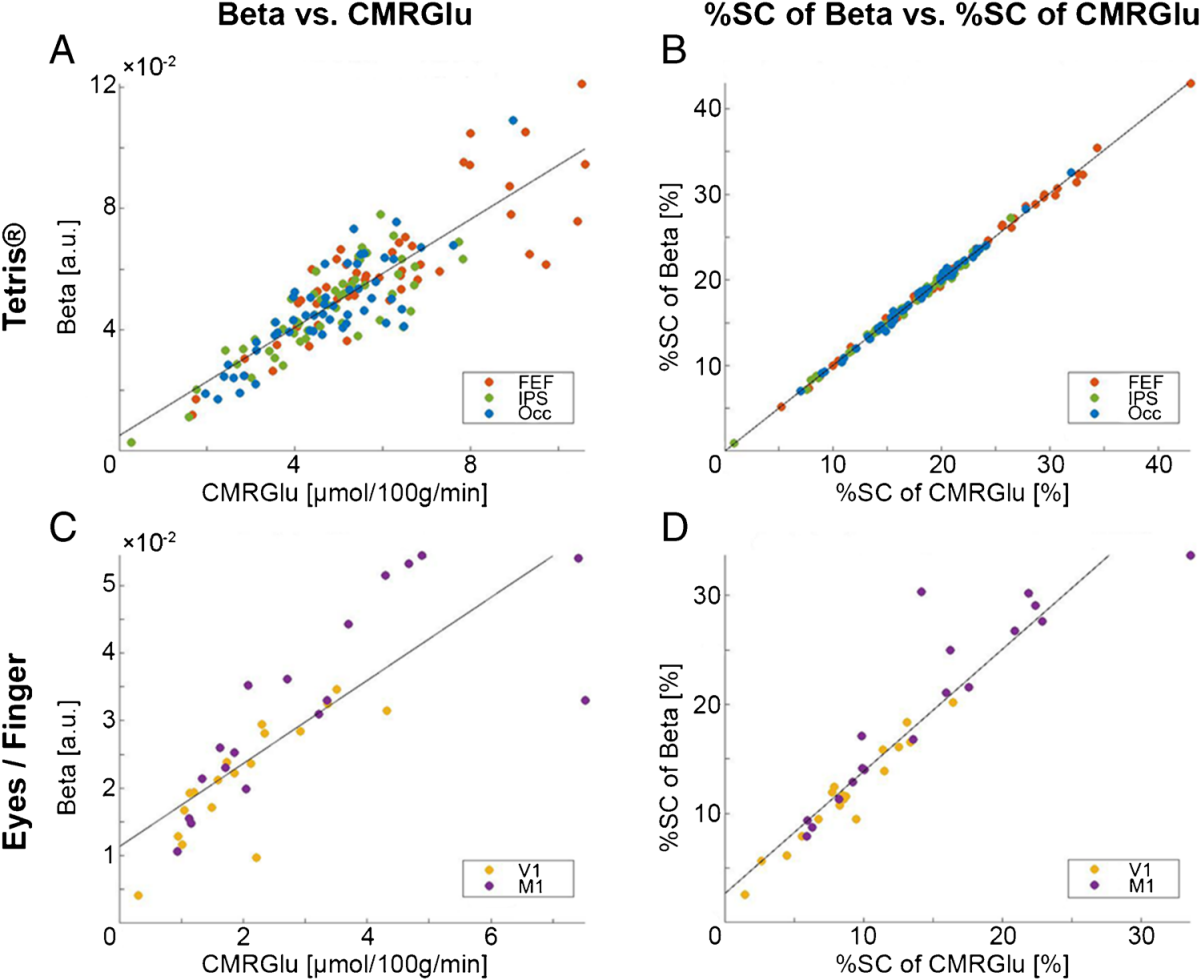
Agreement between outcome parameters. Results of the regression analysis to assess whether beta values (obtained from the general linear model) are correlated with the cerebral metabolic rate of glucose (CMRGlu) across all participants. This was done for beta and CMRGlu values (**A**, **C**), as well as for their percent signal change (%SC) values (**B**, **D**). The figure compares these sets of analysis for task “hard”, for the Tetris®-dataset (DS1, **A**, **B**), and eye opening as well as right finger-tapping for the second dataset (DS2, **C**, **D**). For DS1, the frontal eye field (FEF), the intraparietal sulcus (IPS) and the secondary occipital cortex (Occ) were considered as regions of interest [16]. DS2 displayed activation in the primary visual cortex (V1) for eye opening and the primary motor cortex (M1) for finger-tapping [14]

Our proposed fPET technique differs from previous approaches in one important aspect, namely its independence from an input function in general, be it arterial, venous, image-derived, or population-based. The strong correlation between %SC of task beta estimates and %SC of CMRGlu (i.e., *r* > 0.998, slope ~ 1, intercept ~ 0, Table 1, Fig. 3) and the congruence of activation patterns across different parameters (Fig. 2) validates the non-invasive approach as a robust alternative for performing fPET. This suggests that the underlying theoretical framework is consistent with the experimental data. Moreover, our approach appears to be suitable for tasks of different complexity, such as the demanding Tetris® paradigm and the simpler visual and finger tapping tasks. The slightly reduced performance in DS2 likely reflects lower SNR due to the constant infusion of the radiotracer, without the initial bolus. This may also explain the non-zero intercept in the regression lines of the infusion protocol. Nevertheless, the use of %SC is advantageous for participants as it eliminates the need for arterial cannulation and simplifies experimental procedures. This may be particularly valuable in clinical settings, where resources are often limited and procedural complexity should be minimized. Consequently, the adoption of %SC enhances the applicability of fPET in clinical environments and opens up new possibilities for diagnostic procedures beyond static PET imaging in patient cohorts [20].

However, it is important to acknowledge certain limitations of the simplified fPET approach (Table 2). The obtained metabolic changes are relative to a baseline condition when using %SC of betas as outcome parameter. Moreover, absolute quantification is not possible, neither for task nor baseline effects. Therefore, the technique is only suitable when the precise identification of baseline metabolism and absolute quantification are of no interest. On the other hand, the baseline definition itself becomes critical as different baselines (e.g., eyes closed, eyes open, crosshair fixation, etc.) will result in a different %SC. This reflects a similar situation encountered in fMRI, where the contrast of interest (compared to baseline or a control task) determines increases or decreases in activation [21, 22]. Furthermore, there is a monotonic increase of the signal over time due to the inherent property of [^18^F]FDG to remain mostly trapped in the cell. Therefore, not only the definition but also the timing of the baseline acquisition becomes relevant. To ensure robust modeling, it is recommended to acquire baseline periods in the beginning and end of the scan as well as between tasks [7].

**Table 2:** Visual representation of the three outcome parameters and their main features. The table displays several key features of the main outcome parameters, as obtained by [^18^F]FDG fPET and analysis with the general linear model (GLM). The parameters include the beta maps as output of the GLM, the percent signal change (%SC) of beta estimates (see Eq. (5)) and the gold standard cerebral metabolic rate of glucose (CMRGlu). The general availability of a feature for a certain outcome parameter is marked by a tick, advantages are indicated by a plus sign and disadvantages by a minus sign

Feature	Beta estimate	%SC of beta	CMRGlu
Identification of overall task activation	✓	✓	✓
Identification of individual effects		✓	✓
Applicable for tasks of different complexity	✓	✓	✓
Absolute quantification			+
Quantification of baseline metabolism			+
Influence of baseline definition		-	
Input function required			-

Regarding the use of plain beta estimates (without additional computation of %SC), it is important to note that the agreement with CMRGlu across individuals was generally lower for task effects, and poor for baseline metabolism (Fig. 3, Table 1). Despite this, group-level activations still exhibited high similarity (Fig. 2), suggesting that this outcome parameter may only be used to identify overall activation patterns. However, %SC only requires minimal computational effort and is thus preferable, particularly if individual values are to be related to other metrics of behavior or disease progression. Interestingly, a previous study has reported task-induced signal changes of approximately 2% [4], in contrast to 20–30% observed in this work (Fig. 3B, D). However, their %SC was calculated only as a ratio of plain betas and using the same ratio for our data would result in changes in a similar range of approximately 3% (DS1). This discrepancy (and presumably also the lower agreement with CMRGlu) arises from the fact that beta values alone can be compared across participants only if the underlying regressors are identical. For this reason, we computed %SC from the slope of the product of beta*regressor (see Eq. (5)).

The match between different outcome parameters at the individual level (Supplementary Fig. 3) is evident from the fact that these represent scaled versions of the plain beta estimates (i.e., scaled by the AIF or baseline metabolism, Fig. 1). Though, this scaling is different between subjects, eventually leading to a decreased agreement (Fig. 3). Together, this implies that for individual evaluation any outcome parameter can be used as long as this is done within a subject. However, when comparisons are drawn between subjects, CMRGlu and %SC are more reliable parameters than plain beta estimates.

Although the use of fPET %SC as a proxy of neuronal activation may at first glance appear similar to BOLD fMRI, several essential differences should be kept in mind. The BOLD signal is a composite signal derived not only from neuronal oxygen consumption but also from variations in cerebral blood flow and volume [23], while glucose metabolism is a more direct measure of synaptic activity [24, 25]. Furthermore, fPET is independent of cerebral blood flow, as demonstrated by hypercapnia experiments [2]. Thus, BOLD fMRI and [^18^F]FDG fPET capture complementary aspects of neuronal activation, as demonstrated by task-evoked dissociations between the two parameters in the default mode network [8, 17, 26]. Another significant distinction lies in the test-retest variability of the methods. Previous work has indicated higher reliability for fPET than for fMRI [15, 27]. This variability implies that robust fMRI disease markers are difficult to establish [28], which contributes to the rare use in clinical routine. In contrast, fPET seems to be a promising approach to compare intra-individual changes over time or group comparisons between imaging sites. Moreover, the technique might be relevant to assess changes in neuronal activation as induced by more potent stimulations, such as pharmacological interventions and brain stimulations. For the latter, fPET may hold additional promise since the magnetic field of the MRI limits its compatibility with various electrical devices.

Conventional [^18^F]FDG brain scans are commonly applied for differential diagnosis in cognitive impairment, dementia, movement disorders, presurgical assessment of epilepsy, encephalitis, and neuro-oncology [20]. Unlike current [^18^F]FDG scans, which mostly utilize the standardized uptake value (SUV), non-invasive fPET offers additional stimulation-specific information with a semi-quantitative clinically comparable measure (%SC), while still allowing for the calculation of baseline SUV. An additional benefit lies in the standardized application protocol for fPET. Currently, the time between tracer application and the actual scan as well as the missing control over the behavioral state (e.g., eyes open/closed, etc.) is a source of variability that limits comparability between scans. Furthermore, the assessment of stimulation-induced metabolism in clinical routine would allow characterization of specific alterations of brain function, i.e., working memory in dementia, audiometry for cochlear implants, speech, and motor tasks for preoperative mapping in epilepsy and diagnosis of movement disorders, respectively. Although absolute quantification with arterial blood sampling will continue to be of importance for certain questions, non-invasive fPET may be the preferable alternative in several clinical and research settings, as arterial cannulation will not be tolerated in all patient groups (e.g., children, elderly [8], etc.) and not all research settings have the resources to perform arterial sampling.

A specific limitation of the current study lies in the relatively smaller sample size of DS2 (*n* = 18 vs. *n* = 52). While the retrospective nature of the analysis restricts the possibility of equalizing the sample, the inability to compare with other studies arises from the unique aspect of our validation involving arterial blood sampling, a methodology not employed in other datasets. Nonetheless, we would like to note that the overall size of both datasets exceeds the typical range for other methodological validations in the field [2, 3, 6].

Although we showed that sex does not affect the agreement between the different outcome parameters, the limited age range of the participants in this study (18–35 years) does not allow the investigation of age effects. Nevertheless, the mathematical approximation above indicates that such variables should theoretically not compromise the agreement.

## Conclusions

Our results suggest that plain beta estimates from the GLM may only be suitable when the overall group-averaged activation pattern is to be identified. However, computing %SC of beta values only requires minimal additional effort and represents a valid parameter to study task activation with fPET. Our data further indicates that the introduced approach is generalizable across cognitive domains and load. Still, differences between tasks may occur, which should be considered when defining the baseline condition or using control tasks for comparison. Finally, if absolute CMRGlu and baseline metabolism are of interest, full quantification is required. In sum, assessing task-specific changes in glucose metabolism with %SC is a simple and robust approach that eliminates the need for potentially painful and resource-intensive arterial blood sampling, thereby increasing the accessibility of the technique. The removal of barriers could facilitate the integration of fPET into clinical settings, where arterial blood sampling has traditionally been a major limitation.

### Supplementary Information

Below is the link to the electronic supplementary material.

Supplementary file1 (DOCX 2271 KB)

## Data Availability

Raw data will not be publicly available due to reasons of data protection. Processed data and custom code can be obtained from the corresponding author with a data-sharing agreement, approved by the departments of legal affairs and data clearing of the Medical University of Vienna.
