# Supplementary material for: Non-invasive assessment of stimulation-specific changes in cerebral glucose metabolism with functional PET
Source: Eur J Nucl Med Mol Imaging. 2024 Mar 16;51(8):2283–92. doi: 10.1007/s00259-024-06675-0 (PMC11178598; doi:10.1007/s00259-024-06675-0)
Supplement: Supplementary file 1 — Supplementary file1 (DOCX 2271 KB) [file 259_2024_6675_MOESM1_ESM.docx]

# SUPPLEMENTARY MATERIAL FOR

# Non-invasive assessment of

# stimulation-specific changes in cerebral glucose metabolism with functional PET

Godber M Godbersen^1,2^, Pia Falb^1,2^, Sebastian Klug^1,2^, Leo R Silberbauer^1,2^, Murray B Reed^1,2^, Lukas Nics^3^, Marcus Hacker^3^, Rupert Lanzenberger^1,2#^, Andreas Hahn^1,2#^

*^1^ Department of Psychiatry and Psychotherapy, Medical University of Vienna, Vienna, Austria*

*^2^ Comprehensive Center for Clinical Neurosciences and Mental Health (C3NMH), Medical University of Vienna, Vienna, Austria*

*^3^ Department of Biomedical Imaging and Image-guided Therapy, Division of Nuclear Medicine, Medical University of Vienna, Vienna, Austria*

**^#^ Correspondence to:** Rupert Lanzenberger, Prof. PD MD

Email: [rupert.lanzenberger@meduniwien.ac.at](mailto:rupert.lanzenberger@meduniwien.ac.at)

or

Andreas Hahn, Assoc.Prof. PD PhD MSc

Email: [andreas.hahn@meduniwien.ac.at](mailto:andreas.hahn@meduniwien.ac.at)

Department of Psychiatry and Psychotherapy

Medical University of Vienna, Austria

Waehringer Guertel 18-20, 1090 Vienna, Austria

# MATERIALS AND METHODS

## A detailed description of the respective studies’ procedures can be found in our previous works [1,2], and the timings of task performance relative to fPET data acquisition are shown in Supplementary figure 1.

## Experimental design and tasks

The experimental design of DS1 consisted of a T1-weighted structural MRI and subsequent fPET/fMRI acquisition (52 min). After an initial baseline period (8 min), participants completed four separate sessions of Tetris® with a duration of six minutes each (2x easy and 2x hard level of difficulty, randomized). After each task block, an additional resting period of five minutes was scheduled. The task for DS1 was a modified version of Tetris®. The levels of difficulty were defined by the speed of the falling bricks and the already existing number of bricks at the start of each round. During the task, participants were able to control the game with their right hand only. All participants underwent an initial training, in which they practiced the task in both difficulties for 30 seconds each, before the scan. All periods of rest were defined by participants looking at a crosshair and letting their thoughts wander freely. Regarding DS1, only the data of the first PET/MR measurement [3], was analyzed in this work.

For the collection of the DS2, participants were examined in a 95-minute-long fPET/fMRI scan, which included four separate ten-minute blocks of task performance. Between each of these task blocks, a 15-minute period of rest was scheduled. At the beginning of the scan, a T1-weighted structural image was recorded. The four blocks of task performance consisted of participants opening their eyes at 10-20 and 60-70 min as well as tapping their right thumb to their fingers at 35-45 and 85-95 min after the beginning of the radiotracer administration. During the initial baseline and the periods of rest, participants had their eyes closed and did not move their fingers.

## Participants

For both datasets, participants underwent medical pre-examinations as well as the Structural Clinical Interview for DSM-IV, led by an experienced psychiatrist. These also included an assessment of the general state of health, blood laboratory tests, electrocardiography and a neurological evaluation. Exclusion criteria were former or current somatic, neurological or psychiatric disorders, former or current substance abuse and medication intake, previous study-related radiation exposure as well as pregnancy or breastfeeding. For DS1, specifically, it was also essential that participants did not have any previous experience playing Tetris® within the past three years.

## PET/MRI data acquisition and processing

DS1 participants were administered with [^18^F]FDG according to a bolus plus constant infusion protocol (510 kBq/kg/frame for 1 min and 40 kBq/kg/frame for 51 min). This was realized by employing a perfusion pump (Syramed µSP6000, Arcomed, Regensdorf, Switzerland). The PET data comprised by DS1 was corrected for attenuation and reconstructed using the ordinary Poisson ordered subset expectation maximization algorithm (OP-OSEM) (3 iterations, 21 subsets) into 30-second frames, with an overall matrix size of 344 x 344 and 127 slices. The structural MRI was recorded by applying a T1-weighted MPRAGE sequence (TE/TR = 4.21/2200 ms, voxel size = 1 x 1 x 1.1 mm, 7.72 min).

For DS2, the radiotracer was administered according to a constant infusion protocol which lasted the entire scan. The dose was 3 MBq/kg bodyweight and the radiotracer was distributed at a speed of 36 ml/h by a pump (Volumed µVP7000, Arcomed, Regensdorf, Switzerland). The PET data was reconstructed into one-minute frames using OP-OSEM and corrected for attenuation via an additional CT or a pseudo-CT [4], calculated from a T1-weighted image, for one participant. The T1-weighted structural image was obtained by a MPRAGE sequence (TE/TR = 4.2/2000 ms, voxel size = 1 x 1 x 1.1 mm).

Data pre-processing of both studies’ fPET data was done using SPM12 and included motion correction (quality = 1, registered to mean), spatial normalization to MNI-space with transformation matrices obtained from the structural MRI and smoothing with an 8 mm Gaussian kernel. Masking of the datasets led to the exclusion of non-gray-matter voxels and low-pass filters were used. The cutoff frequency was set to half of the task duration, i.e., 1/3 min for DS1 and 1/5 min for DS2.

## Blood sampling

For both datasets manual arterial blood samples were collected. For DS1, this was done at 3, 4, 5 min as well as 14, 25, 36, 47 min after the start of the tracer application. For DS2 arterial samples were taken at 10, 20, 35, 45, 60, 70, 85 and 95 min after the start of the radiotracer application. The respective arterial samples of both datasets were analyzed regarding their whole-blood and plasma activity with a γ-counter (Wizward2, 3”, Perkin Elmer), which was cross-calibrated to the PET/MR scanner. This enabled the construction of individual AIFs while correcting for the plasma-to-whole-blood ratio. For DS1, this was realized by linear interpolation [5], while data of DS2 were modeled with the sum of two exponential functions.

## Quantification of CMRGlu

The task regressors were modeled as linear ramp functions with a slope of 1 kBq/frame. In the case of DS1, the baseline regressor was calculated by averaging the time course across all gray matter voxels, but excluding those voxels that were declared as active by the fMRI (p < 0.05 FWE corrected voxel level) [5]. For DS2, the baseline regressor was estimated by averaging the time course across all gray matter voxels, which was modeled by a third order polynomial function while controlling for task effects. Although these approaches slightly differ, we have previously shown that the results are highly comparable and do not affect test-retest reliability [1,5,6]. Finally, the regressor accounting for movement artifacts was defined by the first principal component of the six realignment parameters, for both datasets.

# SUPPLEMENTARY FIGURES


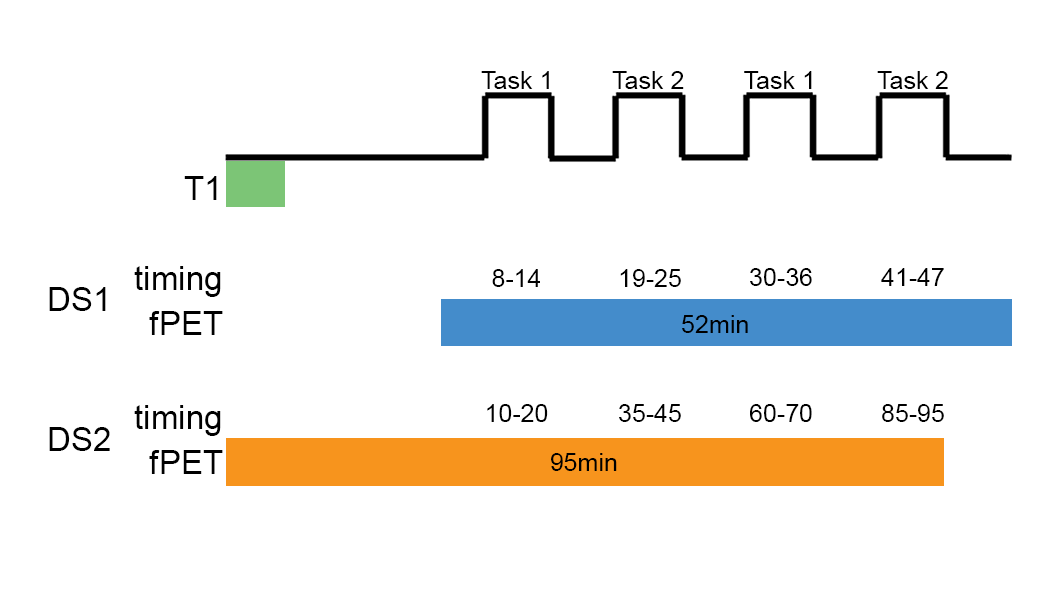


Supplementary figure 1: *Visual representation of the task design for both studies.* A T1-weighted structural MRI was recorded for both studies at the beginning of the measurement. Both data sets (DS) included two tasks, each performed twice. For DS1, the video game Tetris® was performed at two levels of difficulty, easy and hard. For DS2, the tasks consisted of eye opening and tapping the right thumb to the other fingers. The duration of the fPET scan as well as the timing of the tasks relative to radiotracer injection and simultaneous start of the fPET scan are indicated in minutes. Timing differences arise from different radiotracer application with bolus+infusion for DS1 and constant infusion only for DS2. During resting periods, participants either looked at a crosshair, letting their thoughts wander (DS1) or had their eyes closed and did not move their hand (DS2).


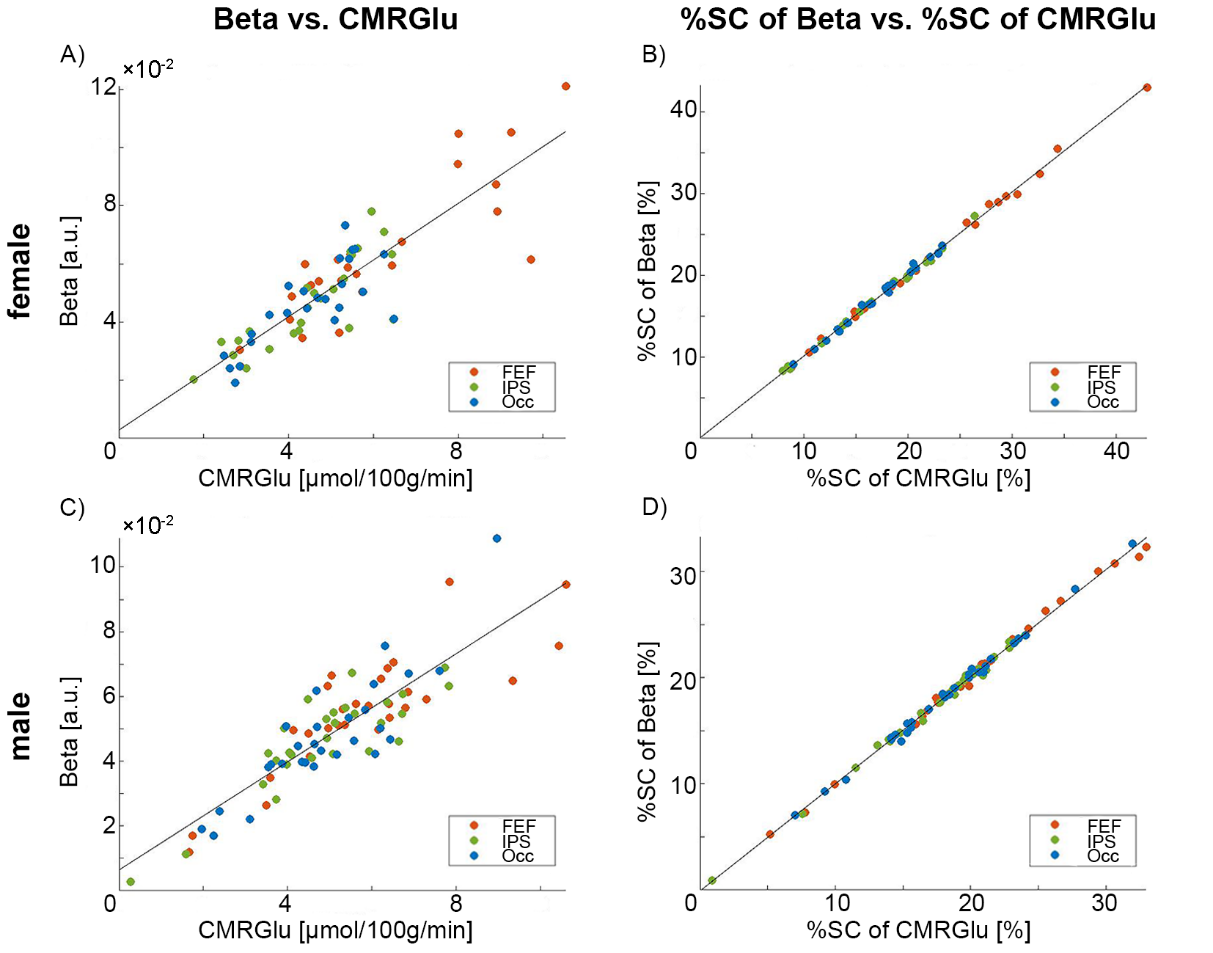


Supplementary figure 2: *Additional analysis of the agreement between outcome parameters separately for women (n=24) and men (n=28) of DS1.* Results of the regression analysis to assess whether beta values (obtained from the general linear model) are correlated with the cerebral metabolic rate of glucose (CMRGlu) across participants, separately for female (A, B) and male (C, D) subgroups. This was done for beta and CMRGlu values (A, C), as well as for their percent signal change (%SC) values (B, D). The figure compares these sets of analysis for task “hard”, for the Tetris®-dataset (DS1). Here, the frontal eye field (FEF), the intraparietal sulcus (IPS) and the secondary occipital cortex (Occ) were considered as region of interest [3].


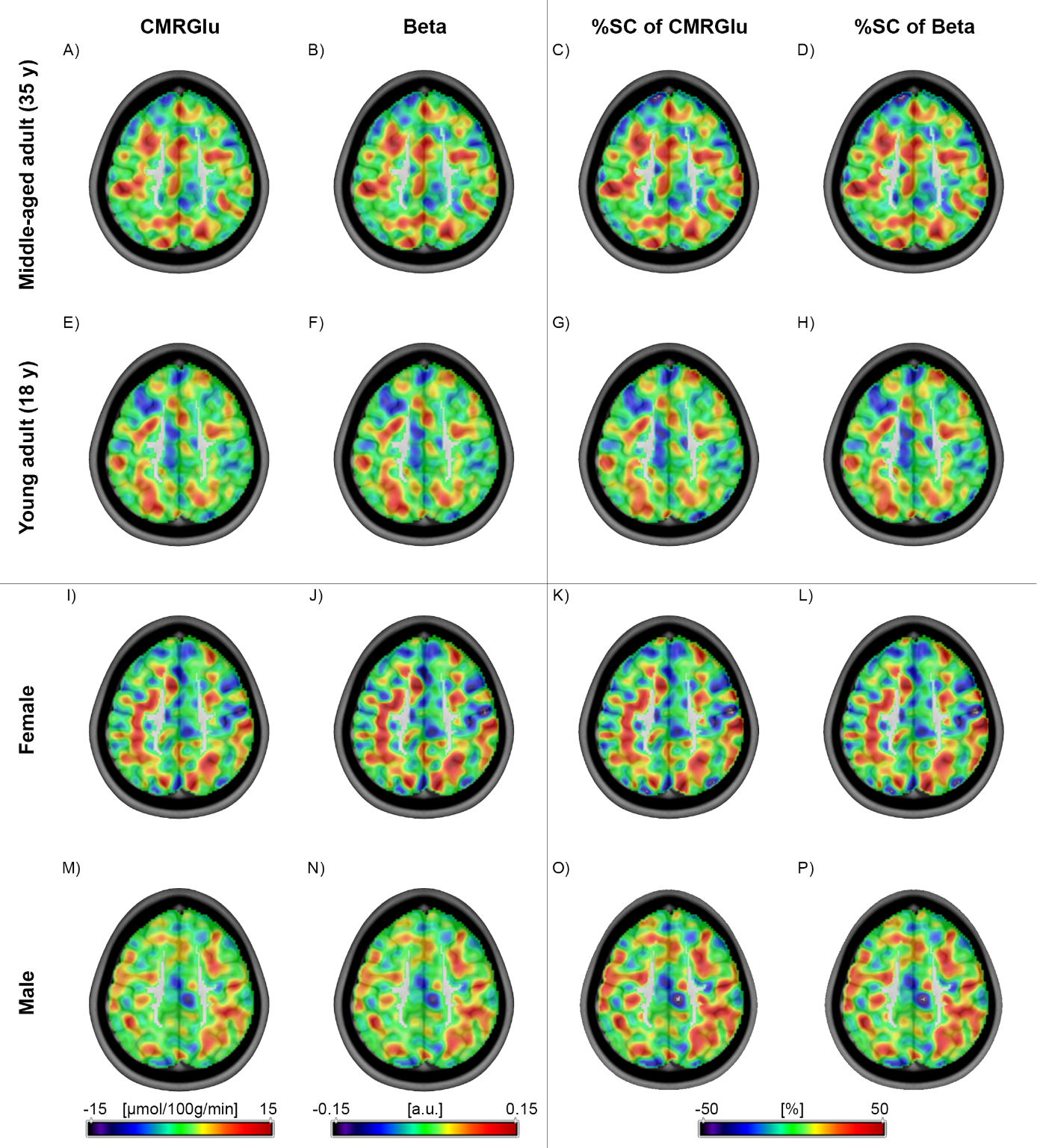


Supplementary figure 3: Comparison of outcome parameters for individual subjects with different demographic features. The figure displays the task-specific effects for DS1 (hard condition). The first two rows (A-H) each show one young (18 years) and one middle-aged adult (35 years) male subject. The two bottom rows (I-P) represent one female and male subject, both of the same age (24 years). Each column represents one outcome parameter: cerebral metabolic rate of glucose (CMRGlu), plain beta maps, and the % signal change (%SC) of both quantities relative to the baseline metabolism. The images highlight the agreement within an individual, which is obvious as the parameters CMRGlu and %SC represent scaled versions of the plain beta estimates (i.e., CMRGlu scaled by the arterial input function (AIF), see Eq. (2), and %SC scaled by baseline metabolism, see Eq. (5) and figure 1). This is because CMRGlu is computed based on the respective Patlak plot that requires the input of plain beta estimates and the AIF, which is consistent within but not between subjects. %SC on the other hand reflects deviations from baseline metabolism, which comprises a multiplication of baseline regressors and the corresponding beta values. The individual difference in task-specific effects, the AIF and the baseline metabolism in turn yields a difference in the outcome parameters between subjects. Axial slices are at z = 50mm MNI space, where also group statistics show activation (see Figure 3). The colorbars illustrate the overall visible range of values which varies between the different quantities.

# SUPPLEMENTARY TABLES

| Sex | Condition |  | ROI |  | Beta vs. CMRGlu | | |  | %SC of Beta vs. %SC of CMRGlu | | |
| --- | --- | --- | --- | --- | --- | --- | --- | --- | --- | --- | --- |
|  |  |  |  |  | R | slope | intercept |  | R | slope | intercept |
| Female | Tetris Easy |  | FEF |  | 0.916 | 0.010 | 0.000 |  | 0.999 | 0.997 | 0.173 |
|  |  |  | IPS |  | 0.870 | 0.010 | 0.001 |  | 0.999 | 0.994 | 0.197 |
|  |  |  | Occ |  | 0.917 | 0.010 | 0.002 |  | 0.999 | 0.995 | 0.167 |
|  | Tetris Hard |  | FEF |  | 0.862 | 0.010 | 0.004 |  | 0.999 | 1.001 | 0.135 |
|  |  |  | IPS |  | 0.820 | 0.009 | 0.003 |  | 0.998 | 1.001 | 0.110 |
|  |  |  | Occ |  | 0.782 | 0.009 | 0.005 |  | 0.997 | 1.018 | -0.149 |
| Male | Tetris Easy |  | FEF |  | 0.924 | 0.008 | 0.009 |  | 0.999 | 0.997 | 0.105 |
|  |  |  | IPS |  | 0.889 | 0.007 | 0.010 |  | 0.998 | 1.011 | -0.126 |
|  |  |  | Occ |  | 0.915 | 0.010 | 0.000 |  | 0.999 | 1.015 | -0.171 |
|  | Tetris Hard |  | FEF |  | 0.856 | 0.008 | 0.011 |  | 0.998 | 0.999 | 0.092 |
|  |  |  | IPS |  | 0.862 | 0.008 | 0.009 |  | 0.998 | 1.007 | -0.073 |
|  |  |  | Occ |  | 0.879 | 0.010 | -0.003 |  | 0.998 | 1.026 | -0.400 |

Supplementary table 1: *Agreement between different quantification methods, separately for female and male participants*. The table shows the results of correlation and regression analyses conducted for the first dataset (DS1), separately with regard to the subjects’ sex. Comparisons were performed for two different levels, either relating the general linear model beta values to the respective cerebral metabolic rate of glucose (CMRGlu, left) or the percent signal change (%SC) of both quantities with each other (right). DS1 comprised three regions of interest (ROI): the frontal eye field (FEF), intraparietal sulcus (IPS) and occipital cortex (Occ) [3].

# REFERENCES

1. Hahn A, Breakspear M, Rischka L, Wadsak W, Godbersen GM, Pichler V, et al. Reconfiguration of functional brain networks and metabolic cost converge during task performance. eLife. eLife Sciences Publications, Ltd; 2020;9.

2. Hahn A, Gryglewski G, Nics L, Rischka L, Ganger S, Sigurdardottir H, et al. Task-relevant brain networks identified with simultaneous PET/MR imaging of metabolism and connectivity. Brain Structure and Function. Springer Berlin Heidelberg; 2018;223:1369–78.

3. Klug S, Godbersen GM, Rischka L, Wadsak W, Pichler V, Klöbl M, et al. Learning induces coordinated neuronal plasticity of metabolic demands and functional brain networks. Communications Biology. Nature Publishing Group; 2022;5:428.

4. Burgos N, Cardoso MJ, Thielemans K, Modat M, Pedemonte S, Dickson J, et al. Attenuation correction synthesis for hybrid PET-MR scanners: Application to brain studies. IEEE Transactions on Medical Imaging. 2014;33:2332–41.

5. Rischka L, Gryglewski G, Pfaff S, Vanicek T, Hienert M, Klöbl M, et al. Reduced task durations in functional PET imaging with [18F]FDG approaching that of functional MRI. NeuroImage. 2018;181:323–30.

6. Rischka L, Godbersen GM, Pichler V, Michenthaler P, Klug S, Klöbl M, et al. Reliability of task-specific neuronal activation assessed with functional PET, ASL and BOLD imaging. Journal of Cerebral Blood Flow and Metabolism. SAGE PublicationsSage UK: London, England; 2021;41:2986–99.
